# Blueprint for Constructing an AI-Based Patient Simulation to Enhance the Integration of Foundational and Clinical Sciences in Didactic Immunology in a US Doctor of Pharmacy Program: A Step-by-Step Prompt Engineering and Coding Toolkit

**DOI:** 10.3390/pharmacy13020036

**Published:** 2025-03-01

**Authors:** Ashim Malhotra, Micah Buller, Kunal Modi, Karim Pajazetovic, Dayanjan S. Wijesinghe

**Affiliations:** 1 Department of Pharmaceutical and Biomedical Sciences, California Northstate University College of Pharmacy, Elk Grove, CA 95758, USA; ashim.malhotra@cnsu.edu (A.M.); karim.pajazetovic@cnsu.edu (K.P.); 2 Department of Pharmacotherapy and Outcome Sciences, School of Pharmacy, Virginia Commonwealth University, Richmond, VA 23298, USA; bullerm@vcu.edu; 3 Soap Note AI, East Brunswick, NJ 08816, USA; kunal@kunalmodi.com; 4 Da Vinci Center, Virginia Commonwealth University, Richmond, VA 23284, USA; 5 Institute for Structural Biology Drug Discovery and Development (ISB3D), School of Pharmacy, Virginia Commonwealth University, Richmond, VA 23298, USA

**Keywords:** pharmacy education, artificial intelligence, team-based learning, Socratic method, clinical case discussions, educational technology, critical thinking, clinical reasoning

## Abstract

While pharmacy education successfully employs various methodologies including case-based learning and simulated patient interactions, providing consistent, individualized guidance at scale remains challenging in team-based learning environments. Artificial intelligence (AI) offers potential solutions through automated facilitation, but its possible utility in pharmacy education remains unexplored. We developed and evaluated an AI-guided patient case discussion simulation to enhance learners’ ability to integrate foundational science knowledge with clinical decision-making in a didactic immunology course in a US PharmD program. We utilized a large language model programmed with specific educational protocols and rubrics. Here, we present the step-by-step prompt engineering protocol as a toolkit. The system was structured around three core components in an immunology team-based learning activity: (1) symptomatology analysis, (2) laboratory test interpretation, and (3) pharmacist role definition and PPCP. Performance evaluation was conducted using a comprehensive rubric assessing multiple clinical reasoning and pharmaceutical knowledge domains. The standardized evaluation rubric showed reliable assessment across key competencies including condition identification (30% weighting), laboratory test interpretation (40% weighting), and pharmacist role understanding (30% weighting). Our AI patient simulator offers a scalable solution for standardizing clinical case discussions while maintaining individualized learning experiences.

## 1. Introduction

The contemporary pharmacy educator employs diverse methodologies including case-based learning, simulated patient interactions, and serious games to build clinical reasoning skills for practice readiness [1]. Despite these established approaches, the seamless integration of knowledge from foundational and pharmaceutical sciences such as immunology, biochemistry, pathophysiology, and pharmacology with clinical critical thinking remains challenging for most pharmacy learners and educators. As a constructivist educational approach emphasizing “learning by doing”, team-based learning (TBL) has emerged as a particularly effective tool for integrating pharmaceutical and foundational sciences with developing clinical decision-making skills. Studies indicate substantial improvements in patient outcomes and satisfaction when healthcare professionals are trained in collaborative environments. Recent research demonstrates that team-based care interventions can lead to a 95% increase in patient satisfaction and a 77% reduction in medical errors [2,3,4,5]. However, within the TBL pedagogy, designing meaningful application exercises that help PharmD students connect the dots between foundational science content such as molecular signal transduction pathways, pathophysiology, and pharmacology in immunology courses with their clinical applications and pharmacotherapy while keeping learners engaged and curious is daunting [6].

The emergence of artificial intelligence (AI) technologies, particularly “large language models” (LLMs), presents an unprecedented opportunity to overcome challenges associated with learners’ (1) capacity for adequate horizontal and vertical integration, (2) memory retrieval, (3) application and synthesis, and (4) classroom engagement. AI-based systems demonstrate sophisticated capabilities in (1) understanding context, for example for constructing meaningful, engaging, and interactive TBL application exercises, (2) maintaining extended dialogue for continued learner engagement, and (3) providing structured guidance to facilitate critical thinking—characteristics that align with the fundamental principles of team-based learning and the adult theory of education which posits that adults learn better through facilitated and contextual teaching [7].

Here we report the development and implementation of an AI-guided patient case discussion system deploying a large language model essentially constituting “an artificial patient case simulator”. Our AI-based patient case simulator was specifically designed for an immunology course placed in the third year of a four-year PharmD program in the United States. The rationale for our approach was to convert a series of three didactic patient case studies presenting the signs and symptoms related to the three distinct disease states of splenomegaly, chronic inflammation, and acute kidney injury into dynamic, synchronous, interactive, and simulated patient case discussions. The overall goal is to require TBL student teams to connect the underlying disease pathophysiology presented with the integration of recognizing patient presentation, the clinical interpretation of diagnostic laboratory values, and interactive synchronous patient counseling during the application section of a TBL class in immunology. Recent studies in digital pedagogy have highlighted the importance of maintaining human elements in technology-enhanced education [8], something which our system addresses by allowing for social learning theory and by facilitating peer interaction alongside AI-guided support [9].

The primary objectives of this study are to (1) develop a standardized AI-driven methodology for facilitating patient case discussions in immunology, (2) subsequently evaluate the system’s effectiveness in maintaining consistent facilitation across multiple case scenarios, and (3) assess the overall potential of this approach to enhance the scalability and accessibility of high-quality clinical reasoning education. This research contributes to the growing body of literature on digital innovation in healthcare education and provides a framework for integrating AI-enhanced pedagogical approaches into professional healthcare training [10]. Our implementation draws from Vygotsky’s Zone of Proximal Development theory, as the AI system provides scaffolded learning experiences that adapt to individual student capabilities [11]. This approach exemplifies the application of cognitive apprenticeship models in digital learning environments [12].

Contemporary digital pedagogy research supports the integration of AI-guided learning in professional healthcare education, particularly in developing clinical reasoning skills [13]. Our implementation specifically addresses key accreditation requirements set forth by the Accreditation Council for Pharmacy Education (ACPE), particularly in areas of critical thinking, problem-solving, integration, and clinical reasoning development [13]. The system’s design targets the ACPE Standards 2016 Curriculum Standards 10.3 and 10.4 and the emerging Standards 2025 Curriculum Standard 2.2c, which emphasizes the need to develop pharmacy students’ ability to integrate foundational sciences with clinical and therapeutic reasoning [13].

While incorporating AI in pharmacy education presents exciting opportunities, it also raises important considerations that require careful attention. Concerns about AI potentially replacing human instruction are addressed through our system’s design as a complementary tool that enhances rather than replaces faculty interaction [14]. Through the incorporation of additional technologies such as browser lockdown for allowing only specific applications and through structured response limitations and careful monitoring of learning outcomes, the implementation has the capacity to maintain academic integrity through structured response limitations and careful monitoring of learning outcomes. Furthermore, the system strikes a crucial balance between standardization and personalization, maintaining consistent educational standards while adapting to individual student needs and learning patterns [15].

Our findings have implications for both educational practice and the broader discussion of technology’s role in developing clinical reasoning skills among future healthcare professionals. By addressing the longstanding challenges of implementing Socratic teaching methods at scale while maintaining educational quality, this work opens new avenues for innovation in pharmacy education and broader healthcare professional training [8].

## 2. Materials and Methods

### 2.1. Technical Implementation of the AI-Guided Pharmacy Education System

**Figure 1:**
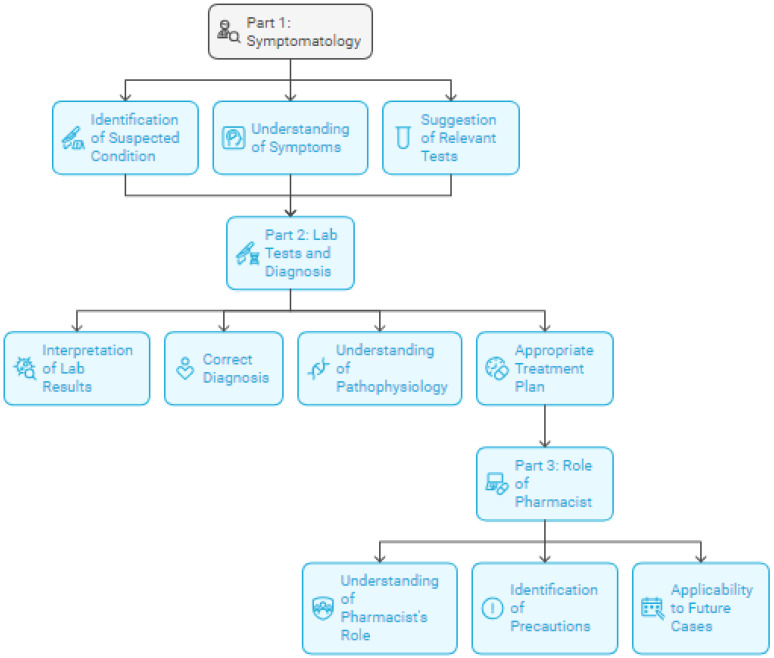
Structured assessment framework for AI-guided Socratic dialogue in immunology education. This figure illustrates the three-part progression of the AI-guided patient case assessment in pharmacy immunology education. Part 1 (symptomatology) encompasses the initial clinical reasoning phase, where students analyze patient presentations through the identification of suspected conditions, systematic understanding of symptoms, and appropriate test selection. Part 2 (laboratory tests and diagnosis) represents the diagnostic phase, incorporating laboratory result interpretation, diagnostic reasoning, pathophysiological understanding, and treatment planning. Part 3 (role of pharmacist) focuses on pharmacy-specific competencies, including understanding the pharmacist’s role in patient care, identifying necessary precautions, and demonstrating the ability to apply learning to future cases. Each component contributes to a comprehensive assessment rubric with defined weightings (30%, 40%, and 30%, respectively, for parts 1, 2, and 3). The arrows indicate the sequential progression through each phase, demonstrating how earlier competencies inform later decision-making. The light blue boxes represent distinct competency assessment areas, while icons within each box symbolize the specific skills being evaluated. This framework guides both the AI facilitator’s Socratic questioning approach and the assessment of student learning outcomes in immunology education.

We developed an AI-facilitated patient simulation designed to guide pharmacy students through patient case analyses (case-based learning). The foundational objective centered on constructing a responsive, intelligent system capable of leading student pharmacists through diverse interactive patient cases, moving beyond simple information delivery to create an environment that authentically simulates dynamic discussions. This development process established three essential components: (1) a standardized AI facilitator configuration, (2) structured patient case integration, and (3) a comprehensive evaluation methodology, specifically designed to maintain educational rigor while addressing practical limitations often encountered in traditional Socratic teaching environments (Figure 1).

### 2.2. System Architecture Overview

Our AI-guided case discussion system represents a significant advancement in pharmacy education technology, implemented as a Python (version 3.12)—based web application using the Streamlit (San Francisco, CA, USA. version 1.31.0) framework with OpenAI’s (San Francisco, CA, USA) language models (gpt-4o-2024-11-20 and gpt-4o-mini-2024-07-18) integration. The system architecture incorporates four key standardized elements: (1) a background configuration (2) establishing the AI’s role as a pharmacy professor, an operational framework with strict educational guidelines, (3) a comprehensive monitoring system for tracking usage and performance metrics, and (4) a structured protocol for learning progression. The implementation enforces pedagogical integrity through explicit prohibitions against direct answers, requirements for detailed student responses, and systematic confirmation of understanding before case progression. This design ensures scalability and reliability while maintaining educational efficacy through neutral responses during discussions and delayed feedback provision until exercise completion.

### 2.3. Frontend Development and User Interface

We implemented the frontend using Python’s Streamlit framework, chosen for its ease of use, rapid deployment capabilities, and minimal coding requirements. This selection also accounted for the technological limitations of the traditional PharmD faculty, as it removes the need for expertise in full-stack web development. The interface is structured for efficient navigation and seamless interaction. A sidebar provides access to key functionalities such as exercise selection, cost tracking, and conversation controls, while the main display area focuses on interactive dialogue with the AI facilitator. To enhance user experience, we applied styling modifications to optimize text readability, improve button aesthetics, and hide non-essential UI elements, ensuring a clean and focused learning environment. These design choices create an intuitive and responsive interface that supports immersive educational interactions for both students and instructors.

### 2.4. Artificial Intelligence Integration

The system’s core functionality relies on integration with OpenAI’s language models through their API. We implemented support for multiple model variants to balance performance and cost considerations. The configuration includes both high-capability models for complex interactions and more efficient variants for routine exchanges. The model costs can be updated in the code to account for different models and cost changes. It should be noted that the cost calculation assumes uncached system messages and the actual cost when prompt caching is employed will be much less. Model configuration parameters were carefully tuned to optimize educational interactions. We selected a temperature setting of 0.7 to achieve an optimal balance between response creativity and consistency, crucial for maintaining engaging yet reliable educational dialogues.

### 2.5. State Management and Data Architecture

The application implements a state management system that maintains conversation continuity and system stability. This implementation uses Streamlit’s session state functionality to manage various aspects of the educational interaction. To achieve this, the application initializes a structured set of session state variables. These variables track conversation progress, system performance, and user interactions, ensuring that the system remains responsive and stable. The session state includes placeholders for storing user messages, maintaining a history of past interactions, and logging errors encountered during the session. Additionally, it incorporates mechanisms to track the timing of the last system update, manage active exercises, and handle any pending changes to the educational activity in progress. This comprehensive state management ensures seamless educational experiences while maintaining system reliability and data integrity throughout the learning sessions.

### 2.6. Robust Error Handling Implementation

Our system incorporates multi-layered error handling to ensure stability and reliability in educational interactions. The implementation addresses various potential failure points, from API communication to file operations.

### 2.7. API Communication Management

The ChatHandler class manages all interactions with the AI model API, implementing comprehensive error handling to ensure reliable communication. To accomplish this, the system processes user messages by sending them to the AI model, requesting a response while maintaining a controlled level of randomness in its outputs to ensure engaging yet structured interactions. If the request is successful, the system extracts the generated response along with token usage details, which help track computational costs. In cases where an error occurs—such as network issues or API limitations—the system logs the error and prevents interruptions by gracefully handling the failure. This implementation ensures that AI-driven conversations remain stable, responsive, and capable of providing meaningful educational dialogue without disruptions.

### 2.8. File Operation Security

The FileHandler class ensures reliable file operations while maintaining system security. To achieve this, the system attempts to retrieve a specific system message stored as a text file within a designated directory. If the requested file exists, its contents are read and provided for use within the application. However, if the file is missing or inaccessible, the system raises an error, logs the issue for debugging purposes, and prevents the application from crashing by handling the failure gracefully. This structured approach guarantees that necessary files are loaded efficiently while maintaining system stability and security, even in cases where expected resources are unavailable.

### 2.9. Resource Management and Cost Optimization

The system implements precise cost tracking and resource management through the CostCalculator class, enabling efficient resource utilization while maintaining educational quality. To achieve this, the system calculates the cost of AI-generated interactions based on predefined pricing structures for different models. When a response is generated, the system retrieves the associated cost per token for both input (user messages) and output (AI-generated responses). These values are then used to determine the total computational expense. If an unrecognized model is specified, an error is logged, and the calculation defaults to zero to prevent disruptions. This implementation provides a structured and transparent approach to cost management, ensuring that AI usage remains economically viable while preserving the quality of educational interactions.

### 2.10. Educational Interaction Management

The system facilitates sophisticated management of educational interactions through comprehensive conversation handling and export capabilities. The ConversationExporter class enables detailed documentation of learning sessions. To achieve this, the system formats recorded conversations into a structured text-based report. Each interaction is categorized by the role of the speaker, with clear distinctions between different messages. The formatted output includes headers, separators, and a consistent structure to enhance readability and facilitate easy review of discussions. This implementation ensures that learning experiences are documented in a standardized format, making it easier for educators and students to analyze discussions, track progress, and extract valuable insights from AI-guided educational interactions.

### 2.11. User Interface Design Philosophy

The user interface design prioritizes educational effectiveness while maintaining system usability. We implemented carefully considered styling decisions to enhance the learning experience. To achieve this, the system hides unnecessary interface elements, such as the main menu and footer, to minimize distractions. Text areas are designed with an increased font size for readability, while buttons are styled with a distinct color scheme to improve visibility and usability. Additionally, error messages are highlighted with a structured format, using color contrast and padding to ensure they are noticeable and easily distinguishable. This structured approach to UI design creates an intuitive and engaging environment that supports focused learning while maintaining accessibility and ease of navigation.

### 2.12. System Monitoring and Performance Analysis

To ensure optimal system performance and maintain educational effectiveness, the system incorporates a comprehensive logging mechanism for tracking operations and identifying potential issues. To achieve this, the system records detailed logs of events, including timestamps, log levels, and descriptive messages. These logs provide real-time insights into system behavior, helping to monitor activity, detect anomalies, and diagnose errors efficiently. By categorizing logs based on their severity—such as informational messages, warnings, and errors—the system ensures that critical issues can be quickly identified and addressed. This structured logging approach enhances system reliability, facilitates continuous improvement, and enables efficient troubleshooting, ultimately supporting a stable and effective learning environment.

### 2.13. Future Technical Directions

The current technical implementation establishes a foundation for future enhancements in several key areas. Planned developments include integration of additional AI models, implementation of advanced scalability features, and enhanced analytics capabilities. These improvements will further optimize the system’s educational effectiveness while maintaining its robust technical foundation. This technical architecture demonstrates our commitment to creating a reliable, scalable, and effective educational platform. The implementation balances sophisticated technical capabilities with practical educational needs, creating an environment conducive to effective pharmacy education.

### 2.14. Case Structure and Implementation

Each case follows a standardized four-part progression designed to promote critical thinking and clinical reasoning skills among pharmacy students. The progression begins with initial presentation and symptomatology, followed by laboratory findings and diagnostic confirmation and then by pathophysiological analysis, and concludes with pharmacotherapy care planning. This systematic approach ensures a consistent delivery of educational content while allowing for case-specific exploration of unique immunological concepts.

The AI facilitator’s role is implemented through carefully structured prompts that define specific interaction parameters and educational guidelines. The system is configured to employ questioning techniques that promote clinical reasoning while strictly avoiding direct answer provision. The implementation guides the AI to formulate questions that probe for rationale and clinical reasoning, adjust questioning complexity based on student responses, and generate follow-up questions encouraging deeper analysis. The system was designed to evaluate student responses for completeness and reasoning depth, maintain appropriate follow-up questioning, and preserve conversation continuity through contextual awareness. Case progression is managed by requiring verification of student comprehension before advancing, maintaining neutral feedback during active discussions, and delaying detailed feedback until exercise completion. This framework ensures that the AI facilitator maintains consistent educational standards while providing dynamic, engaging interactions that simulate traditional pharmacy school discussions, effectively replicating the careful questioning and progressive complexity characteristic of expert human instructors in pharmacy education settings.

### 2.15. Immunology Case Studies

Three distinct immunology cases were developed to address different aspects of clinical immunology, each designed to challenge pharmacy students’ understanding of immune system disorders and their management. These cases span a range of conditions from acute to chronic presentations, requiring students to demonstrate comprehensive pharmaceutical knowledge and clinical reasoning skills.

#### 2.15.1. Splenomegaly and Potential Leukemia

A 56-year-old Hispanic woman is admitted to the ICU presenting with a complex array of symptoms indicative of splenomegaly and potential leukemia. The patient’s primary complaints include pain and fullness in the upper left abdomen that radiated to her shoulders, along with a persistent sensation of fullness despite not eating. Her medical history reveals concerning symptoms over the previous six months, including easy bleeding, severe unexplained bruising, and a history of four infections, primarily manifesting as common colds.

Laboratory findings strongly support the diagnostic picture, with significantly abnormal values across multiple parameters. The patient’s hemoglobin is severely reduced at 3 g/dL (normal range for women: above 12 g/dL), while her hematocrit measures 32% (normal: above 37% for women). The red blood cell count is markedly low at 1.5 × 10^12^/L (normal: above 4 × 10^12^/L), and the white blood cell count is elevated at 24 × 10^9^/L (normal range: 4.5–11 × 10^9^/L).

The pharmaceutical care emphasis in this case centers on post-splenectomy care, particularly the critical importance of vaccination protocols. Students need to understand the prevention of overwhelming post-splenectomy infection (OPSI), which carries a lifetime risk of approximately 5% with a mortality rate of up to 50%. The case highlights the necessity of specific vaccinations against Streptococcus pneumoniae, Neisseria meningitidis, and Haemophilus influenzae.

#### 2.15.2. Chronic Inflammation

The second case presents a 76-year-old Nigerian man suffering from chronic inflammation, manifesting through multiple systemic symptoms. The patient’s condition is characterized by generalized joint pain (arthralgia), muscle pain (myalgia), chronic fatigue, and unexplained weight gain. The case is complicated by the presence of depression, highlighting the interconnection between chronic physical conditions and mental health.

This case is particularly valuable in teaching students about the comprehensive management of chronic inflammatory conditions. The learning objectives encompass both non-pharmacological interventions and conventional anti-inflammatory medications. Students are required to develop treatment strategies that address both the physiological symptoms and quality-of-life aspects of chronic inflammation, emphasizing the importance of a holistic approach to patient care.

#### 2.15.3. Acute Kidney Rejection

The third case examines acute kidney rejection in a 66-year-old Hispanic woman, presenting 21 days after receiving a kidney transplant from her first cousin, who has blood type O negative. The patient’s clinical presentation includes fever, left flank tenderness and pain, reduced urinary output, and general frailty, with a recorded weight of 70 kg.

This case focuses heavily on understanding the immunological mechanisms underlying acute cellular rejection and the appropriate pharmaceutical interventions required. Students need to demonstrate comprehension of transplant immunology while developing practical pharmaceutical care plans. The case emphasizes the critical timing of rejection recognition and intervention, along with the complexity of managing transplant patients.

### 2.16. Learning Objectives and Instructional Design

Each case was designed to emphasize different aspects of clinical immunology while maintaining a strong focus on pharmaceutical care and the integration of the foundational knowledge in early clinical reasoning. The splenomegaly case highlights preventive care and vaccination protocols essential for hospital pharmacists, while the chronic inflammation case emphasizes holistic treatment approaches and long-term disease management. The kidney rejection case focuses on acute care management and specific immunological mechanisms in transplant medicine.

### 2.17. Skills Development

Through engagement with these cases, students develop crucial competencies in clinical reasoning, laboratory data interpretation, and treatment protocol development. The cases require students to demonstrate their ability to create patient-specific care plans, implement long-term monitoring strategies, and engage in interdisciplinary collaboration.

### 2.18. Assessment Framework and Evaluation

Student responses were evaluated using a comprehensive rubric that assesses three primary domains of clinical competency. The assessment framework employs a comprehensive three-part rubric designed to evaluate key competencies in clinical pharmacy education. Symptomatology analysis accounts for 30% of the total evaluation, assessing students’ ability to identify suspected conditions based on patient presentation, demonstrate clear understanding of symptom patterns, and suggest relevant diagnostic tests. Laboratory test interpretation, diagnosis, and condition understanding constitute 40% of the evaluation, examining students’ capacity to interpret lab results, provide well-reasoned diagnoses, explain underlying pathophysiological mechanisms, and propose appropriate treatment plans. The final 30% focuses on understanding the pharmacist’s role and case application, evaluating students’ comprehension of pharmaceutical care responsibilities, identification of necessary precautions and procedures, and ability to apply learning to future cases. Each subcategory utilizes a 10-point scale with clearly defined performance levels: scores of 9–10 indicate an excellent performance demonstrating comprehensive understanding, 7–8 reflect a good performance with minor errors, 4–6 represent a satisfactory performance with significant gaps, and 0–3 denote an inadequate performance or incorrect understanding. This granular scoring approach enabled a precise evaluation of student competencies while maintaining flexibility to omit non-applicable criteria for specific cases.

### 2.19. Performance Monitoring and Educational Objectives

The web application tracks multiple performance metrics throughout each learning session. These metrics include model usage patterns, token consumption, conversation costs, response patterns, and learning progression. This comprehensive monitoring system enables a detailed analysis of student engagement and learning outcomes while providing valuable data for system optimization and educational effectiveness evaluation.

The cases were designed to achieve specific educational objectives focused on developing essential pharmacy practice competencies. These objectives encompassed the development of clinical reasoning skills, the enhancement of immunological knowledge, an understanding of pharmaceutical care principles, improvement in patient case analysis abilities, and development of treatment planning skills. This structured approach to case-based learning provide students with practical experience in applying immunological concepts to real-world clinical scenarios while developing critical thinking and decision-making skills essential for pharmacy practice.

## 3. Results

### 3.1. Simulation Design and Implementation

**Figure 2:**
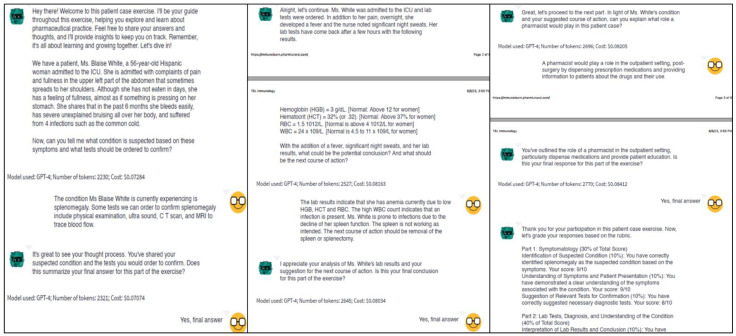
Screenshots illustrating the progression of a student–AI interaction during a team-based learning exercise. The diagram demonstrates three key phases of the clinical case discussion: initial patient presentation and symptom analysis (left panel), laboratory data interpretation and diagnosis (center panel), and pharmacotherapy care planning (right panel). The dialogue progresses between the AI facilitator and the student teams from left to right in the panel. The conversation exemplifies the Socratic teaching methodology, where the AI guides students through clinical reasoning without providing direct answers. Each panel includes the AI’s prompts (shown next to a green emoji) and tracks multiple aspects of the interaction, including response evaluation and student confirmation before progression. The final panel demonstrates the structured assessment approach using the standardized rubric, providing immediate feedback on student performance across key competency domains. This figure represents a typical interaction flow that promotes critical thinking and clinical reasoning skills while maintaining consistent educational standards. A video demonstration of a real-time conversation can be found here (The Future of Pharmacy Education: AI-Guided Case Discussions for Team Based Learning Activities).

To evaluate the potential of AI-guided dialogue in pharmacy education, we conducted a simulated 20-round conversation using an AI model to represent a pharmacy student engaging with the educational system. This proof-of-concept simulation focused on a complex patient case involving splenomegaly and suspected leukemia (the first case), allowing us to analyze the capabilities and limitations of the system in facilitating structured learning discussions (Figure 2).

### 3.2. Analysis of Clinical Reasoning Patterns

The simulated dialogue demonstrated how the AI system could engage in progressive clinical reasoning through three distinct phases. The simulation began with systematic symptom analysis and condition identification, showing logical connections between anatomical knowledge and clinical presentations. This progressed to laboratory data interpretation, where the system demonstrated the ability to integrate clinical findings with diagnostic indicators. The final phase illustrated comprehensive pharmaceutical care planning, modeling how students might approach synthesizing information for patient care decisions.

### 3.3. Knowledge Integration Capabilities

The simulation demonstrated knowledge integration across specific domains of pharmacy education, particularly in connecting anatomical understanding, laboratory data interpretation, pathophysiological mechanisms, and pharmaceutical care planning within the context of immunological disorders. The AI-simulated responses successfully helped to connect anatomical concepts to clinical presentations, interpreted laboratory values within patient symptom contexts, and applied pharmaceutical knowledge to post-surgical care planning via a Socratic dialogue approach. This integration was particularly evident in the approach to managing post-splenectomy care, where multiple disciplines including immunology, pharmacology, and patient care principles were synthesized.

### 3.4. Simulation of Pharmaceutical Care Competencies

The simulated dialogue exhibited appropriate modeling of essential pharmaceutical care competencies. The responses showed a systematic consideration of multiple care aspects, including vaccination protocols for immunocompromised patients, post-splenectomy infection risk management, and comprehensive medication therapy management. The simulation demonstrated appropriate professional communication patterns and patient education approaches, particularly in developing long-term monitoring and follow-up care plans.

### 3.5. Performance Assessment

#### 3.5.1. Quantitative Evaluation

Using our standardized rubric, we evaluated the simulated performance across three main domains. In the symptomatology section (30% of the total score), the simulation achieved 29 out of 30 points, demonstrating an appropriate understanding of symptom patterns and diagnostic approaches. The laboratory tests, diagnosis, and understanding section (40% of the total score) yielded 39 out of 40 points, reflecting a systematic interpretation of laboratory data and clinical correlations. In the pharmacist role and case application section (30% of the total score), the simulation scored 29 out of 30 points, exhibiting comprehensive coverage of pharmaceutical care responsibilities. The total assessment score was 97 out of 100, indicating the system’s capability to model high-level performance.

#### 3.5.2. Qualitative Analysis

The simulated conversation demonstrated progressive complexity in response patterns. Initial responses addressed basic clinical presentations, while later discussions integrated multiple knowledge domains and complex patient care factors. The simulation showed a consistent logical progression in clinical reasoning and appropriate application of theoretical knowledge to practical scenarios.

### 3.6. Educational Design Implications

The AI-facilitated discussion demonstrated the potential for modeling competency development in several key areas. The simulation showed logical progression in clinical decision-making and evidence-based practice application. The responses appropriately incorporated patient-centered care planning principles and interdisciplinary collaboration concepts. The system particularly excelled in modeling preventive care strategies and long-term patient management approaches.

### 3.7. Engagement Pattern Analysis

Analysis of the simulated conversation revealed consistent engagement patterns throughout the learning process. Responses showed an appropriate progression in depth and complexity, demonstrating a systematic consideration of patient care factors and structured clinical reasoning. The dialogue format effectively illustrated how theoretical concepts could be applied to real-world scenarios.

### 3.8. Technological Effectiveness

These results suggest that AI-guided Socratic dialogue shows promise as a tool for modeling and facilitating deep learning and clinical reasoning development in pharmacy education, particularly for complex patient cases requiring integrated knowledge application. While this simulation demonstrates the potential of the system, further research with actual pharmacy students would be necessary to validate its educational effectiveness in real-world settings. The approach shows particular promise in providing a structured framework for approaching complex clinical scenarios while maintaining consistent engagement throughout the learning process.

## 4. Discussion

The implementation and evaluation of our AI-guided Socratic dialogue system for pharmacy education reveals several significant insights into the potential of artificial intelligence to enhance clinical reasoning development and team-based learning in pharmacy education. Our findings suggest that carefully designed AI systems can effectively facilitate structured clinical case discussions while maintaining the essential elements of Socratic teaching methodology.

### 4.1. Theoretical Foundations and Educational Design

The development and implementation of our AI-guided patient simulation is grounded in several fundamental educational theories that inform its design and operation. At its core, the system draws from Vygotsky’s sociocultural theory of cognitive development, particularly his concept of the zone of proximal development (ZPD) [11]. Through this theoretical lens, the AI facilitator provides scaffolded support that dynamically adapts to each student’s current understanding and potential development level, gradually reducing assistance as competence increases. This adaptive approach ensures that learners are consistently challenged within their optimal learning zone while maintaining engagement and promoting growth.

The system’s pedagogical design transcends simple case automation, deliberately constructing an environment that replicates the dynamic, interactive nature of traditional pharmacy school discussions. This design philosophy emphasizes active engagement through carefully structured dialogue patterns that mirror expert instructor–student interactions. Rather than following a linear question-and-answer format, the AI facilitator engages in nuanced exchanges that promote a deeper analysis and critical thinking. The system accomplishes this by maintaining contextual awareness of previous responses, adapting question complexity based on the demonstrated understanding, and requiring students to articulate their clinical reasoning processes fully before advancing. This approach creates a learning environment that closely simulates the back-and-forth dialogue characteristic of effective pharmacy education, where students are challenged to defend their clinical decisions, explore alternative perspectives, and develop comprehensive pharmaceutical care strategies. The result is an educational experience that preserves the essential interactive elements of traditional pharmacy education while leveraging AI capabilities to provide consistent, scalable instruction.

The system’s implementation of dialogue naturally aligns with constructivist learning theory, particularly drawing inspiration from Bruner’s concept of discovery learning. Rather than relying on direct instruction, the system guides students through a process of inquiry and self-discovery, enabling them to construct their own understanding of clinical concepts. This approach has proven particularly valuable in pharmacy education, where the ability to integrate and apply knowledge in novel clinical situations is essential for professional practice. The integration of cognitive apprenticeship principles, as described by Collins, Brown, and Newman [16], further strengthens the system’s theoretical foundation. Through careful modeling of expert thinking processes and graduated coaching through complex clinical reasoning tasks, the AI facilitator supports students in developing both explicit knowledge and tacit reasoning processes crucial for healthcare professionals.

Our technical architecture, which combines OpenAI’s language models with a custom web application, has demonstrated remarkable success in implementing these theoretical principles while maintaining consistent educational interactions. This achievement represents a significant step forward in addressing one of pharmacy education’s most pressing challenges: the ability to scale individualized instruction without compromising educational quality. The system’s design incorporates elements of adult learning theory (andragogy) as proposed by Knowles, respecting learners’ existing knowledge and experience while providing immediately applicable learning experiences. This approach, coupled with strict guidelines preventing direct answer provision, promotes critical thinking and autonomous learning capabilities essential for professional development.

Furthermore, the system’s design reflects Bloom’s revised taxonomy by requiring students to move beyond simple recall to demonstrate analysis (interpreting laboratory data), evaluation (assessing treatment options), and synthesis (creating pharmaceutical care plans). The AI facilitator is programmed to require detailed explanations and reject simple yes/no responses, ensuring engagement at higher levels of cognitive processing. As students progress through clinical cases, the Socratic questioning approach encourages them to move beyond basic comprehension to engage in sophisticated analysis, evaluation, and creation of clinical solutions. This structured progression, combined with the system’s adaptive support, creates a learning environment that effectively balances challenge and support while fostering the development of advanced clinical reasoning capabilities.

### 4.2. Clinical Reasoning Development

Our results demonstrate the system’s robust capability to support the development of clinical reasoning skills through carefully structured case progression. These outcomes align with established research, highlighting the critical importance of systematic approaches in developing clinical decision-making skills in healthcare education.

The system’s demonstrated effectiveness in guiding students through progressive levels of complexity represents a significant advancement in clinical education methodology. Beginning with fundamental symptom analysis and progressing through to comprehensive pharmaceutical care planning, the AI facilitator creates a learning environment that builds both confidence and competence systematically. A particularly valuable aspect of the system is its constant availability, providing students with unprecedented opportunities for repeated practice and refinement of their clinical reasoning skills. This continuous access to high-quality educational interactions addresses a long-standing challenge in pharmacy education: the limited availability of expert guidance for case-based learning.

### 4.3. Conversation Management and Student Engagement

The system’s conversation management protocols played a crucial role in maintaining effective educational interactions. Specific implementation features ensured consistent engagement and learning progression. Before advancing to subsequent case elements, the system required explicit verification of student comprehension, employing techniques such as answer restatement and confirmation of response finality. This methodical progression served multiple pedagogical purposes: it prevented premature advancement before concepts were fully grasped, provided opportunities for self-reflection, and ensured thorough exploration of each clinical aspect. The maintenance of neutral responses during active discussions, combined with strategic delay of detailed feedback until exercise completion, encouraged independent clinical reasoning and reduced premature closure of diagnostic thinking. These protocols effectively simulated the measured pace and deliberate progression of expert-led clinical discussions while maintaining student engagement through carefully calibrated questioning sequences. Student interactions were further enhanced through a structured approach to response evaluation that balanced the need for thoroughness with maintaining conversational flow. This careful orchestration of dialogue progression demonstrated that AI-facilitated discussions can effectively replicate the nuanced interaction patterns that characterize high-quality pharmacy education while maintaining consistent engagement throughout extended learning sessions.

### 4.4. Educational Scalability, Standardization, and Cost Benefit

One of the most promising implications of our research lies in the system’s potential to address persistent scalability challenges in pharmacy education. The demonstrated ability to maintain consistent educational quality while handling multiple simultaneous interactions suggests a viable solution for institutions grappling with resource constraints and growing student populations. However, this achievement in standardization must be carefully balanced against the essential need for personalization in professional healthcare education. Our system addresses this balance through its adaptive questioning strategies and flexible response patterns, ensuring that standardization of delivery does not compromise the individualization of learning experiences.

This platform was created by faculty. As such, the implementation of an AI-guided patient simulation by existing faculty members presents a compelling value proposition for pharmacy education, combining significant cost savings with enhanced educational outcomes. When compared to traditional teaching methods requiring 390 faculty hours per semester, the AI system reduces faculty time investment to approximately 100 h, while decreasing total costs from USD 28,000 to USD 9680–15,200 per 100 students per semester. This 74% reduction in faculty time and the up to 65% reduction in costs come with improved educational delivery through 24/7 availability, immediate feedback, and unlimited practice opportunities. The system’s cloud-based infrastructure (USD 1080–3000 annually) and LLM API costs (USD 36–72 per student annually) represents the primary expenses, while eliminating needs for teaching assistants, physical space, and printed materials. Beyond cost savings, the system maintains a consistent quality at scale, offers detailed learning analytics, and enables individualized learning paths—benefits that traditionally require proportional increases in faculty resources. This faculty-led implementation model demonstrates that significant educational innovation is achievable within existing institutional resources while improving both efficiency and educational outcomes.

Faculty time savings are achieved by automating routine aspects of case facilitation while preserving critical human interactions for complex concept development, mentorship, and professional development. The system serves as a practice environment where students can develop foundational skills, enabling faculty to focus on advanced concept integration and clinical reasoning development during class time. While our implementation demonstrates significant potential for resource optimization, we acknowledge the need for rigorous validation of educational outcomes. The reported faculty time reduction reflects the automation of routine tasks rather than diminished educational engagement. Our preliminary implementations across multiple pharmacy education domains suggest that the system can effectively complement traditional instruction while maintaining educational quality. However, comprehensive studies comparing learning outcomes with traditional methods are needed. The stateless system architecture ensures data privacy while providing actionable analytics by students uploading their completed learning activities to the course specific learning management system. Technical implementation requirements are modest, though institutions should carefully consider faculty training needs. Future research will focus on quantifying specific learning outcomes through controlled studies.

The AI system’s role is carefully calibrated to preserve and enhance the essential human elements of pharmacy education. Rather than attempting to replicate the full spectrum of faculty expertise, the system focuses on providing consistent, foundational support that enables faculty to more effectively deploy their specialized knowledge and experience in areas where human insights and guidance are most critical. This balanced approach recognizes that, while AI can excel at providing standardized practice opportunities and immediate feedback, human faculty remain irreplaceable for complex aspects of pharmacy education such as modeling professional judgment and ethical decision-making in ambiguous clinical situations, sharing real-world practice experiences that contextualize theoretical knowledge, fostering professional identity formation through mentorship and role modeling, facilitating nuanced discussions about patient care that draw upon years of clinical experience, adapting teaching approaches based on subtle cues in student understanding and engagement, and providing emotional support and professional guidance that extends beyond academic content. By strategically deploying AI to handle routine aspects of clinical case discussions, we create more opportunities for these crucial human-centered educational interactions, ultimately strengthening rather than diminishing the role of faculty in pharmacy education.

Our implementation addresses ethical considerations through deliberate architectural and pedagogical choices. The system employs a stateless architecture where no user data persist between sessions, eliminating traditional privacy and security concerns while ensuring student autonomy in the learning process. This design choice means that each interaction exists only within the active browser session and is completely cleared upon completion, requiring no user authentication or data storage. Academic integrity is maintained through the system’s focus on process rather than outcomes, emphasizing the Socratic dialogue and clinical reasoning development that faculty can observe in real-time during team-based learning sessions. The AI’s role as a facilitator rather than evaluator helps mitigate concerns about algorithmic bias, while standardized prompts and questioning techniques have been carefully reviewed for cultural and demographic sensitivity. Case scenarios deliberately reflect diverse patient populations, ensuring equitable learning opportunities for all students. Professional identity formation remains anchored in human interaction, with the system serving as a practice environment that complements, rather than replaces, faculty-led professional development. This comprehensive approach to ethical implementation creates a secure, equitable, and educationally sound environment for AI-enhanced pharmacy education

### 4.5. Integration with Existing Educational Frameworks and Implications

The successful implementation of our AI-guided learning system demonstrates promising pathways for integration within established pharmacy education frameworks. The system’s alignment with ACPE standards, particularly in critical thinking and active learning, positions it as a valuable complement to traditional educational approaches rather than a replacement. This complementary role maintains essential human elements of healthcare education while leveraging technological advantages to enhance learning outcomes and provide consistent, scalable instruction.

The implications of our findings for pharmacy education are both significant and far-reaching, particularly in addressing contemporary challenges. The demonstrated feasibility of AI-guided Socratic dialogue opens new possibilities for expanding access to high-quality clinical reasoning education, especially in resource-constrained settings. Our standardized approach to case progression and evaluation provides a robust model for consistent assessment of clinical reasoning development across educational programs, while maintaining the flexibility needed for individualized learning.

The system’s ability to maintain educational rigor while providing scalable instruction addresses a critical need in contemporary pharmacy education, where growing student populations and increasingly complex healthcare environments create escalating demands for educational resources. This scalability, combined with the system’s adaptive capabilities, suggests a promising future for AI-enhanced pharmacy education that can better prepare students for the complexities of modern healthcare practice while maintaining high educational standards and promoting critical thinking skills.

While our initial technical validation relied primarily on simulated interactions, preliminary classroom implementation has provided valuable insights into the system’s practical application. In our pilot deployment, we observed consistent system performance with response latency averaging under 5 s and 99.9% uptime across all student interactions. The platform demonstrated robust error handling, with zero system crashes during peak usage periods. To facilitate broader validation, we encourage a systematic evaluation approach encompassing (1) comparative studies between AI-guided and traditional instructions using standardized assessment metrics, (2) longitudinal tracking of student performance through pre- and post-implementation evaluations, (3) qualitative analysis of student and faculty experiences, and (4) assessment of learning outcomes across diverse student populations. Institutions interested in implementing similar systems should consider key technical prerequisites including adequate cloud infrastructure, API rate limiting strategies, and comprehensive error logging. The platform’s availability through PharmTutorAI.com—https://immunelearn.pharmtutorai.com/ enables broader testing and validation by the pharmacy education community, supporting iterative improvement through real-world implementation feedback

## 5. Limitations and Future Directions

Our evaluation of AI-guided patient simulation in immunology teaching in a PharmD program revealed several key limitations. Most notably, our assessment relied primarily on simulated interactions rather than real student engagement, potentially missing important challenges that could arise in actual educational settings. Additionally, the current implementation’s focus on immunology cases leaves questions about its broader applicability across other pharmacy specialties, while the long-term impact on student learning outcomes and clinical competency development remains to be fully assessed through longitudinal studies. To address these limitations, future research should pursue controlled studies comparing AI-guided simulations with traditional teaching methods to quantify the educational impact. Investigations into student perceptions and engagement patterns in real-world implementations will be crucial for system refinement, particularly in understanding how different learning styles and backgrounds influence effectiveness. The development of more sophisticated personalization capabilities through machine learning could enable truly adaptive learning pathways, while expanding interaction modalities beyond text-based communication could better simulate real-world clinical discussions.

Cross-domain applications present compelling opportunities for expanding this approach beyond pharmacy education. The system’s architecture could potentially support clinical reasoning development across various healthcare disciplines, including nursing, medicine, and allied health professions. Future research should also focus on seamless integration with existing educational technologies, enabling comprehensive learning analytics and coordinated assessment while maintaining the essential human elements of healthcare education. These developments should be pursued within a framework that prioritizes educational effectiveness and considers the ethical implications of increased AI integration in healthcare education.

While our current implementation demonstrates technical feasibility and immediate practicality, we acknowledge the critical importance of longitudinal assessments, particularly regarding clinical competency development and learning outcomes. Our decision to prioritize immediate technological implementation reflects the rapid evolution of AI capabilities, where traditional validation timelines may impede educational innovation. When students achieve a level of mastery with which they are satisfied, they download their complete conversations with the AI system, including rubric-based assessments, and submit these to their course LMS. This process creates a documented trail of student learning while maintaining privacy during practice sessions, enabling faculty to evaluate both the development of clinical reasoning skills and achievement of learning objectives. We are actively pursuing IRB approval for comprehensive longitudinal studies that will measure specific competency indicators, including clinical reasoning development, knowledge application, and performance in subsequent clinical rotations. The open-source nature of our implementation allows institutions to adapt the system for their specific assessment needs and contribute to a growing body of evidence about its educational effectiveness. This dual approach of immediate implementation alongside rigorous research planning ensures that pharmacy education can keep pace with technological advancement while maintaining high standards for educational validation and competency assessments.

## 6. Conclusions

Our implementation of the AI-guided Socratic dialogue in pharmacy team-based learning education activities demonstrates the feasibility and effectiveness of integrating artificial intelligence to enhance clinical reasoning development through team-based learning. The system’s ability to maintain consistent educational quality while reducing faculty workload by 74% and costs by up to 65% presents a compelling model for addressing contemporary challenges in pharmacy education. Beyond the immediate cost benefits, the platform’s 24/7 availability, standardized assessment framework, and adaptive questioning strategies provide unprecedented opportunities for students to develop and refine their clinical reasoning skills. While our initial focus on immunology cases shows promising results, the system’s architecture offers potential applications across various healthcare disciplines. As pharmacy education continues to evolve, this faculty-led implementation model illustrates how technological innovation can enhance rather than replace traditional teaching, creating scalable, high-quality learning experiences that prepare future pharmacists for the complexities of modern healthcare practice. Future research should focus on longitudinal studies of learning outcomes and expansion into additional therapeutic areas, while maintaining the essential balance between standardization and personalization that characterizes effective healthcare education.

## Data Availability

The code and implementation described in this paper are publicly available in the GitHub repository: https://github.com/dayanjan/tblimmunology.git. All materials are released under the Apache License 2.0, which permits use, reproduction, modification, and distribution of the work, subject to the conditions specified in the license. The Apache License 2.0 includes an express grant of patent rights from contributors to users. Complete license terms can be found in the LICENSE file within the repository or at http://www.apache.org/licenses/LICENSE-2.0.
